
JOURNAL INFORMATION
==============================
NLM Title Abbreviation: ScientificWorldJournal
Journal ID: ScientificWorldJournal
NLM Journal ID: 101131163
Journal ID: TSWJ

The Scientific World Journal

ISSN: 2356-6140
EISSN: 1537-744X
Publisher: Wiley

ARTICLE INFORMATION
==============================
PMCID: PMC6009319
PMID: 29973781
DOI: 10.1100/tsw.2002.7
Article ID: 367030
Article version: 1
Subjects: Short Report

Genetic Models of Aerobic Endurance Running Capacity in Rats

Britton Steven L. *
Koch Lauren Gerard
Functional Genomics Laboratory, Medical College of Ohio, 3035 Arlington Avenue, Toledo, Ohio 43614-5804, USA
*Steven L. Britton: sbritton@mco.edu
Publication date: 2002
Electronic publication date: 2002 Apr 18
Volume: 2
First page: 13
Last page: 14
Copyright: Copyright © 2002 Steven L. Britton and Lauren Gerard Koch.
Copyright year: 2002
License: This is an open access article distributed under the Creative Commons Attribution License, which permits unrestricted use, distribution, and reproduction in any medium, provided the original work is properly cited.
License URL: https://creativecommons.org/licenses/by/3.0/

Funding: National Institutes of Health HL64270

==============================
Advances in Gene Technology: The Genome and Beyond –
Structural Biology for Medicine (Proceedings of the 2002 Miami
*Nature Biotechnology* Winter Symposium)
*TheScientificWorld* 2002, 2(S2), 13–14
ISSN 1532-2246; DOI 10.1100/tsw.2002.7

### GENETIC MODELS OF AEROBIC ENDURANCE RUNNING CAPACITY IN RATS

Steven L. Britton and Lauren Gerard Koch
Functional Genomics Laboratory, Medical College of Ohio, 3035 Arlington Avenue, Toledo, Ohio 43614-5804
sbritton@mco.edu

**INTRODUCTION**. The accumulation of atmospheric oxygen starting about 2 billion years ago was permissive for the evolution of complex aerobic metabolic pathways[1,4]. It is thus axiomatic that aerobic capacity defines both a large part of our biology and a divide in the continuum between health and disease[2]. While heritability studies predict a genetic component that accounts for as much as 70 to 90% of the variation in aerobic capacity[3], the genes causative of the difference between low and high capacity have not been defined. In 1996 we started artificial selection for low and high aerobic treadmill-running capacity in rats. The purpose was to create low-capacity runners (LCR) and high-capacity runners (HCR) that could ultimately be developed into contrasting strains for intrinsic (i.e., untrained) aerobic capacity. Here we report the response to selection across nine generations of divergent artificial selection for aerobic treadmill-running capacity.

**METHOD**. Artificial selection for intrinsic, aerobic, endurance-running capacity was started using the genetically heterogeneous N:NIH stock of rats as a founder population (n = 168). Selection for low and high capacity was based upon distance run to exhaustion on a motorized treadmill using a velocity-ramped running protocol.

**RESULTS**. On average the founder population ran to exhaustion in 355 ± 11 m. Nine generations of selection produced lines that differed in running capacity by 270% with most of the change occurring in the high line (HCR). At generation nine, the LCR ran 230 ± 8 m and the HCR ran 851 ± 36 m at exhaustion.

**DISCUSSION**. The large difference (270%) in capacity between the LCR and HCR demonstrate that these will be useful models for discovering the genes responsible for variation in intrinsic aerobic capacity. Our immediate plan is to increase the intensity of selection to produce LCR and HCR that are even more widely (greater than fivefold) different in running capacity and make these available for study. Long term, we will create at least four inbred lines for both LCR and HCR.

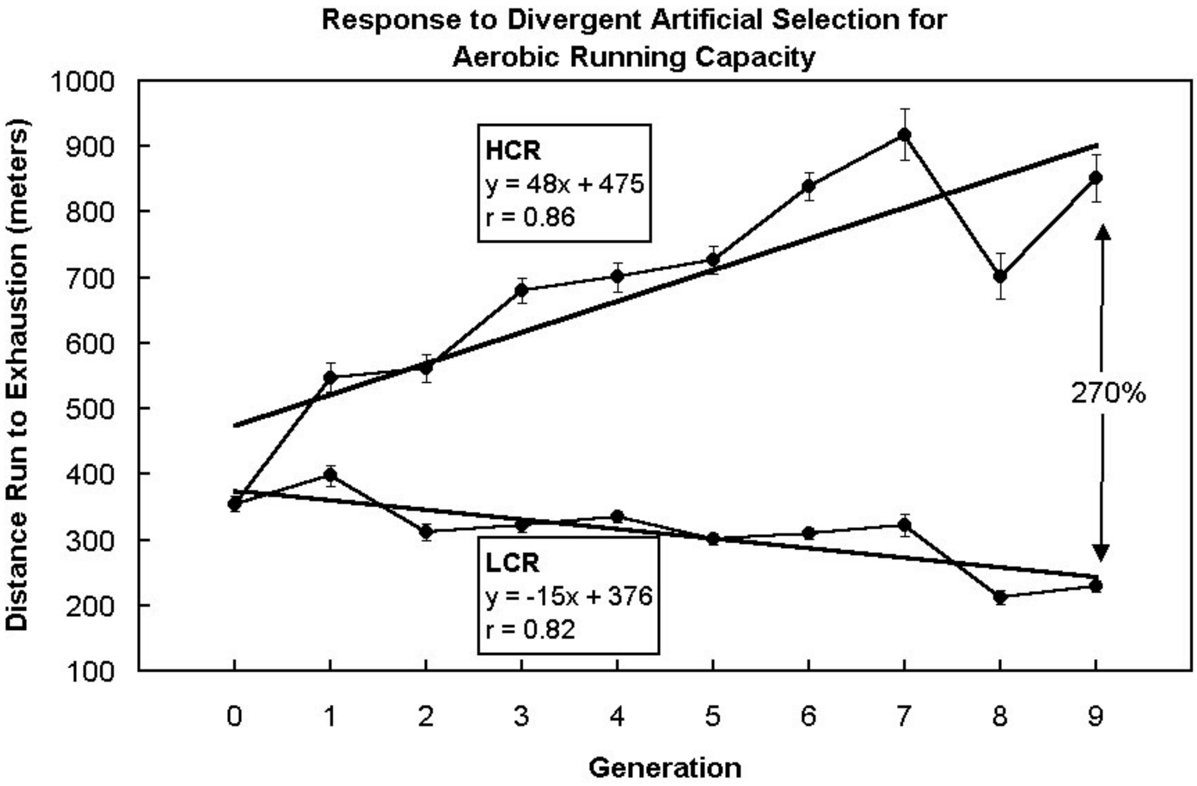

**ACKNOWLEDGEMENTS**. Supported by National Institutes of Health Grant HL64270.

**REFERENCES**

1. Baldwin, J.E. and Krebs, H. (1981) Nature 291, 381–382.
2. Fisher, R.A. (1930) Clarendon Press, Oxford, England.
3. Klissouras, V. (1971) J. Appl. Physiol. 31, 338–344.
4. Paytan, A. (2000) Science 288, 626–627.
